# DUSP1 Is a Novel Target for Enhancing Pancreatic Cancer Cell Sensitivity to Gemcitabine

**DOI:** 10.1371/journal.pone.0084982

**Published:** 2014-01-07

**Authors:** Fang Liu, A. Jesse Gore, Julie L. Wilson, Murray Korc

**Affiliations:** Departments of Medicine, Biochemistry and Molecular Biology, Indiana University School of Medicine, The Melvin and Bren Simon Cancer Center and the Center for Pancreatic Cancer Research, Indianapolis, Indiana, United States of America; University of Colorado Denver, United States of America

## Abstract

Pancreatic ductal adenocarcinoma (PDAC) is a deadly cancer with a poor prognosis that is characterized by excessive mitogenic pathway activation and marked chemoresistance to a broad spectrum of chemotherapeutic drugs. Dual specificity protein phosphatase 1 (DUSP1) is a key negative regulator of mitogen activated protein kinases (MAPKs). Yet, DUSP1 is overexpressed in pancreatic cancer cells (PCCs) in PDAC where it paradoxically enhances colony formation in soft agar and promotes *in vivo* tumorigenicity. However, it is not known whether DUSP1 overexpression contributes to PDAC chemoresistance. Using BxPC3 and COLO-357 human PCCs, we show that gemcitabine activates c-JUN N-terminal kinase (JNK) and p38 mitogen activated protein kinase (p38 MAPK), key kinases in two major stress-activated signaling pathways. Gemcitabine-induced JNK and p38 MAPK activation mediates increased apoptosis, but also transcriptionally upregulates DUSP1, as evidenced by increased DUSP1 mRNA levels and RNA polymerase II loading at DUSP1 gene body. Conversely, shRNA-mediated inhibition of DUSP1 enhances JNK and p38 MAPK activation and gemcitabine chemosensitivity. Using doxycycline-inducible knockdown of DUSP1 in established orthotopic pancreatic tumors, we found that combining gemcitabine with DUSP1 inhibition improves animal survival, attenuates angiogenesis, and enhances apoptotic cell death, as compared with gemcitabine alone. Taken together, these results suggest that gemcitabine-mediated upregulation of DUSP1 contributes to a negative feedback loop that attenuates its beneficial actions on stress pathways and apoptosis, raising the possibility that targeting DUSP1 in PDAC may have the advantage of enhancing gemcitabine chemosensitivity while suppressing angiogenesis.

## Introduction

Pancreatic ductal adenocarcinoma (PDAC) is the fourth leading cause of cancer-related death in the US, with an annual mortality of nearly 38,000, a median survival of 6–7 months and a five-year survival rate of 6% [1]. While resection prolongs survival and offers a potential cure, 80–85% of PDAC are unresectable at the time of diagnosis due to the presence of distant metastases, peritoneal seeding, or invasion into adjacent vital structures [2]. The chemotherapeutic agent gemcitabine (2′,2′-difluorodeoxycytidine, dFdC) has been the standard of care for patients with locally advanced or metastatic disease [3]. Recently, the Food and Drug Administration approved the combination of gemcitabine and nab-paclitaxel, based on the finding that this combination improved overall survival to 8.5 months versus 6.7 months with gemcitabine alone [4]. It is generally accepted that improving responsiveness to gemcitabine in PDAC would lead to an additional increase in patient survival.

The resistance of PDAC to gemcitabine and many other chemotherapeutic agents is due, in part, to a wide range of genetic and epigenetic alterations which lead to abnormal activation of cell survival and anti-apoptotic pathways [5], an intense desmoplasia which interferes with drug delivery to the tumor mass [6], [7], and changes in expression of key molecules involved in gemcitabine uptake, intracellular activation and efflux [8]. There is an urgent need, therefore, to advance our understanding of the mechanisms underlying chemoresistance in PDAC, in order to devise new and more effective chemotherapeutic strategies.

Abnormal activation of mitogen-activated protein kinases (MAPKs) plays a critical role in regulating cell survival and apoptosis [9], [10]. MAPKs can be grouped into three families: extracellular signal-regulated kinase (ERK), c-Jun-NH2 kinase (JNK), and p38 MAPK [9], [10]. Upon stimulation by mitogen or environmental stress, MAPKs are activated through phosphorylation on their tyrosine and threonine residues by upstream MAP2K kinases [9], [10]. Activated MAPKs phosphorylate a spectrum of target substrates, including protein kinases and transcription factors involved in regulating cell proliferation, differentiation, survival, and apoptosis [9], [10]. Despite the existence of crosstalk pathways among different MAPKs, most evidence supports the concept that activated ERK promotes cell proliferation, survival, and motility, while JNKs and p38 MAPKs negatively regulate cell cycle progression and induce apoptotic cell death in response to environmental stress [9], [10].

The dual-specificity phosphatase (DUSP) family of proteins consists of 25 members [11]. DUSPs can dephosphorylate both the threonine/serine and tyrosine residues of their substrates and thus function as negative regulators of MAPKs [11]. DUSP1/MKP-1 is a nuclear MAPK phosphatase that is a direct transcriptional target of p53, E2F-1, c-Jun, and ATF2, and that is induced in response to oxidative stress, hypoxia, and other stresses such as nutritional deprivation and chemotherapeutic drugs [12]–[14]. DUSP1 is overexpressed in a range of epithelial tumors including PDAC, non-small-cell lung cancer, breast, ovarian, gastric, and early-stage prostate cancer [15]–[20], and this overexpression is correlated with poor patient survival in ovarian cancer [18]. The increased DUSP1 expression in breast cancer is inversely correlated with JNK activity and markers of apoptosis, suggesting an anti-apoptotic role of DUSP1 via its activity towards JNK [17]. In support of this conclusion, cancer cells that overexpress DUSP1 are resistant to chemotherapy and Fas ligand-induced apoptosis, whereas reduction of DUSP1 levels using a small interfering RNA enhances sensitivity to these agents [21]–[23]. Conversely, in hepatocellular carcinoma (HCC) increased DUSP1 levels correlate with better prognosis [24]. Moreover, DUSP1 negatively regulates ERK signaling in HCC cells, thereby inhibiting their proliferative potential, suggesting that DUSP1 is a tumor suppressor gene in HCC [24]. Thus, the deleterious consequences of DUSP1 overexpression are likely cancer specific.

We reported that DUSP1 is overexpressed in PDAC, and that antisense-mediated suppression of DUSP1 expression reduces tumor development in nude mice [15]. It is not known, however, whether DUSP1 could be a target for improving response to gemcitabine-based chemotherapy in PDAC. We now demonstrate that gemcitabine activates JNK and p38 MAPK, thereby leading to increased apoptosis and DUSP1 transcription. Conversely, shRNA-mediated inhibition of DUSP1 enhances gemcitabine-induced JNK and p38 MAPK activation and sensitizes PDAC cells to gemcitabine. Using a doxycycline-inducible strategy to suppress DUSP1 in established orthotopic pancreatic tumors, we show that combining gemcitabine with DUSP1 inhibition prolongs survival, attenuates angiogenesis, and enhances apoptotic cell death. Thus, DUSP1 can be a potential therapeutic target for enhancing PDAC sensitivity to gemcitabine.

## Results

### JNK and p38 MAPK Signaling Pathways are Activated in Response to Gemcitabine

**Figure 1:**
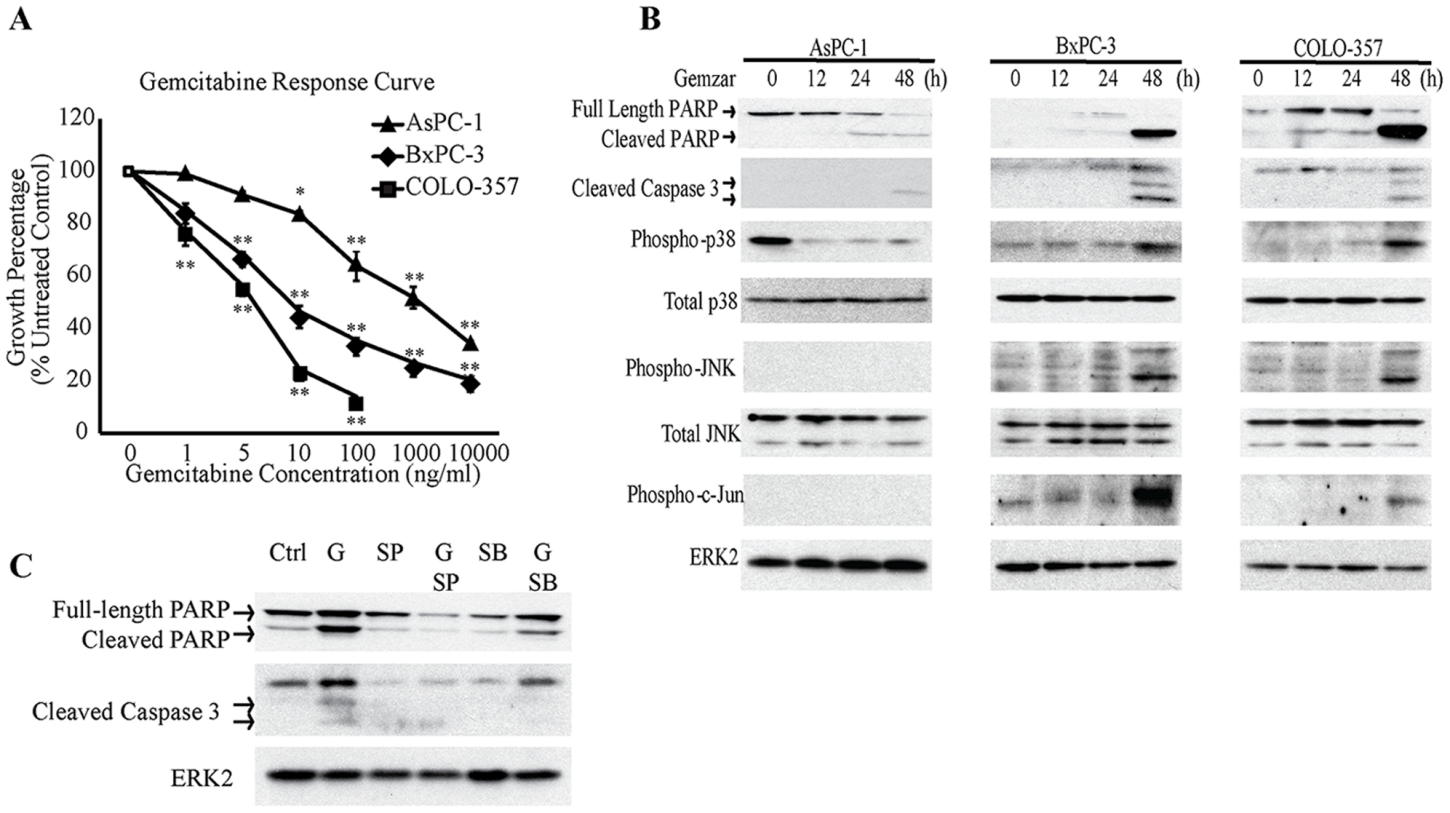
Effects of gemcitabine on human pancreatic cancer cells. (A) AsPC-1, BxPC-3, and COLO-357 cells were incubated for 48 h in the absence or presence of varying concentrations of gemcitabine, and MTT assays were performed. Data are the means ± SEM of 3 experiments. *p<0.05; **p<0.01, compared with control. (B) AsPC-1, BxPC-3, and COLO-357 cells were incubated for the indicated times with 100 ng/ml, 10 ng/ml, and 5 ng/ml gemcitabine, respectively, and analyzed by immunoblotting. (C) BxPC-3 cells were incubated for 48 h with 10 ng/ml gemcitabine (G), in the absence or presence of 10 µM SB203580 (SB) or 10 µM SP600125 (SP), and analyzed by immunoblotting.

The effects of gemcitabine on the PCC growth were evaluated using the MTT assay. In all three cell lines, gemcitabine exerted a dose-dependent growth inhibitory effect. AsPC-1 was most resistant to gemcitabine, with an IC50 greater than 100 ng/mL, whereas BxPC-3 and COLO-357 cells were more sensitive to gemcitabine, with IC50 values of 10 ng/mL and 5 ng/mL, respectively (Fig. 1A).

To evaluate the effects of gemcitabine on the activation of MAPKs, cells were incubated with gemcitabine at a concentration close to their respective IC50 s. In BxPC-3 and COLO-357, gemcitabine induced the phosphorylation of p38 MAPK and JNK, key kinases in two major stress-activated signaling pathways (Fig. 1B). Gemcitabine also increased the levels of phospho-c-Jun in these cells (Fig. 1B). Given that phospho-c-Jun is a common downstream target of p38 MAPK and JNK pathways, this observation confirmed the activation of p38 MAPK and JNK signaling. Moreover, the levels of cleaved caspase 3 and cleaved PARP correlated with p38 MAPK, JNK, and c-Jun activation, suggesting that p38 MAPK and JNK pathways mediate apoptotic cell death in response to gemcitabine (Fig. 1B). By contrast, AsPC-1 cells were more resistant to gemcitabine, as evidenced by the absence of p38 MAPK and JNK activation and the lower levels of cleaved caspase 3 and cleaved PARP, even at a concentration as high as 100 ng/mL (Fig. 1B). To determine whether gemcitabine induced apoptosis through p38 MAPK and JNK activation, BxPC-3 cells were incubated with gemcitabine in the absence or presence of p38 MAPK (SB203580) or JNK (SP600125) inhibitors. The levels of cleaved PARP and cleaved caspase 3 were decreased by SB203580 and SP600125, when added to gemcitabine (Fig. 1C). Thus, in sensitive cell lines, gemcitabine activates p38 MAPK and JNK signaling which induces apoptotic cell death, whereas gemcitabine-resistance is associated with lower levels of apoptosis and markedly attenuated p38 MAPK/JNK activation.

### Gemcitabine Activates DUSP1 Transcription through JNK and p38 MAPK Signaling

**Figure 2:**
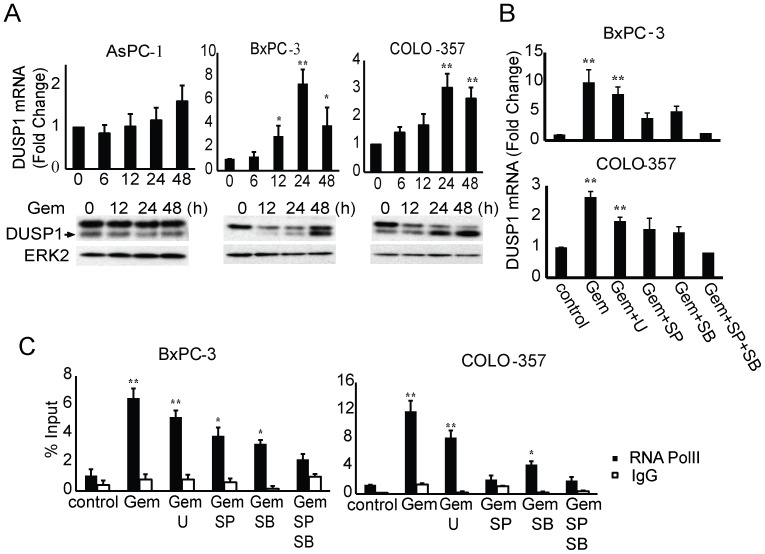
Gemcitabine induces DUSP1 transcription through JNK and p38 MAPK signaling. (A) AsPC-1, BxPC-3, and COLO-357 cells were incubated for the indicated times with 100 ng/ml, 10 ng/ml, and 5 ng/ml gemcitabine, respectively, and DUSP1 levels were assessed by Q-PCR and immunoblotting. (B,C) BxPC-3 and COLO-357 cells were incubated for 24 h with 10 ng/ml and 5 ng/ml gemcitabine, respectively, in the absence or presence of 10 µmol/L U0126, 10 µmol/L SP600125, 10 µmol/L SB203580, or both SP600125 and SB203580, and Q-PCR (B) and RNA polymerase II ChIP followed by Q-PCR for DUSP1 gene body region (C) were performed. All data are the means ± SEM of 3 experiments. *p<0.05; **p<0.01, compared with control.

Considering that DUSP1 is a key regulator of MAPK activities [11], we next examined the expression of DUSP1 in response to gemcitabine and the underlying mechanism regulating its expression. In both BxPC-3 and COLO-357 cells, DUSP1 mRNA and protein levels were induced at 24 h and 48 h following gemcitabine addition, coinciding with the activation of p38 MAPK and JNK (Fig. 2A). By contrast, no significant changes in DUSP1 levels were observed in gemcitabine-resistant AsPC-1 cells, consistent with the low levels of apoptosis and the absence of p38 MAPK and JNK activation upon treatment (Fig. 2A).

In response to various stimuli, the transcription factor AP-1 (c-Jun, c-Fos, ATF2), which is the major downstream target of p38 MAPK and JNK signaling, has been shown to associate with the DUSP1 promoter and regulate its transcription [14], [25]. To determine whether p38 MAPK and JNK signaling mediate the induction of DUSP1 expression by gemcitabine, BxPC-3 and COLO-357 cells were treated with gemcitabine in the absence or presence of SB203580, SP600125, or their combination. SB203580 and SP600125 decreased the induction of DUSP1 mRNA levels by gemcitabine, whereas their combination completely blocked this induction. By contrast, ERK inhibition with U0126 failed to alter gemcitabine-mediated induction of DUSP1, supporting the conclusion that p38 MAPK and JNK rather than ERK signaling mediate DUSP1 induction by gemcitabine (Fig. 2B).

The increase in DUSP1 mRNA levels could be due to enhanced transcriptional activity or posttranscriptional mRNA stability. Therefore, chromatin immunoprecipitation against RNA polymerase II, which is the core component of the transcription machinery, was next carried out, followed by Q-PCR to quantify the amount of RNA polymerase II bound to the gene body region of DUSP1. This assay allows for direct measurement of the active elongation step and reflects the transcriptional activity of the DUSP1 gene. Incubating BxPC-3 and COLO-357 cells with gemcitabine for 24 h increased the amount of RNA polymerase II loading at the gene body region of DUSP1 by 6 fold and 12 fold respectively (Fig. 2C), pointing to transcriptional activation of DUSP1 by gemcitabine. SB203580 and SP600125, but not U0126, inhibited the gemcitabine-induced increase in the amount of RNA polymerase II associated with the DUSP1 gene body (Fig. 2C). Together, these results indicate that p38 MAPK and JNK signaling mediates DUSP1 transcriptional activation by gemcitabine.

### Interruption of the DUSP1 Negative Feedback Loop Enhances Chemosensitivity to Gemcitabine and Cisplatin

**Figure 3:**
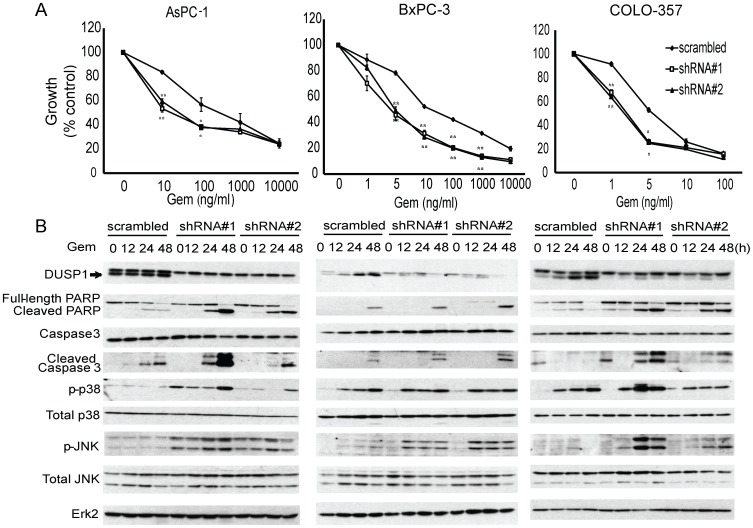
Effects of DUSP1 knockdown on gemcitabine response in human pancreatic cancer cells. AsPC-1, BxPC-3, and COLO-357 cells were stably transduced with lentivirus expressing shRNA against scramble control or DUSP1. (A) Cells were incubated for 48 h in the absence or presence of varying concentrations of gemcitabine, and MTT assays were performed. Data are the means ± SEM of 3 experiments. *p<0.05; **p<0.01, compared with control. (B) AsPC-1, BxPC-3, and COLO-357 cells were incubated for the indicated times with 100 ng/ml, 10 ng/ml, and 5 ng/ml gemcitabine, respectively, and analyzed by immunoblotting.

To investigate the effect of silencing DUSP1 on MAPK activities and gemcitabine chemosensitivity, AsPC-1, BxPC-3, and COLO-357 cells were stably transduced with lentivirus expressing shRNA against DUSP1 or non-targeting scramble control and then incubated with various gemcitabine concentrations. In all three cell lines, DUSP1 knockdown using two shRNAs increased the growth inhibitory actions of gemcitabine. Thus, silencing DUSP1 shifted the IC50 values for gemcitabine in AsPC-1, BxPC-3 and COLO-357 cells from 500 to 10 ng/ml, from 10 to 5 ng/ml, and from to 5 to 2.5 ng/ml, respectively (Fig. 3A). These results suggest that DUSP1 knockdown enhanced gemcitabine chemosensitivity, and that the sensitizing effects were more marked in cells with high chemoresistance.

The anti-DUSP1 antibody routinely revealed 2 closely-migrating bands on western blots (Fig. 2–3). However, DUSP1 knockdown with two highly specific shRNAs targeting DUSP1 specifically silenced expression of the lower band (Fig. 3B), indicating that the upper band was non-specific. DUSP1 knockdown with the same highly specific shRNAs also potentiated gemcitabine-induced apoptosis, as evidenced by increased levels of cleaved PARP and cleaved caspase 3 (Fig. 3B). Higher levels of gemcitabine-induced phospho-p38 MAPK and phospho-JNK were also noted in DUSP1 knockdown cells, compared with scramble control, suggesting that silencing DUSP1 de-repressed p38 MAPK and JNK signaling, thereby leading to enhanced apoptotic cell death in response to gemcitabine (Fig. 3B).

**Figure 4:**
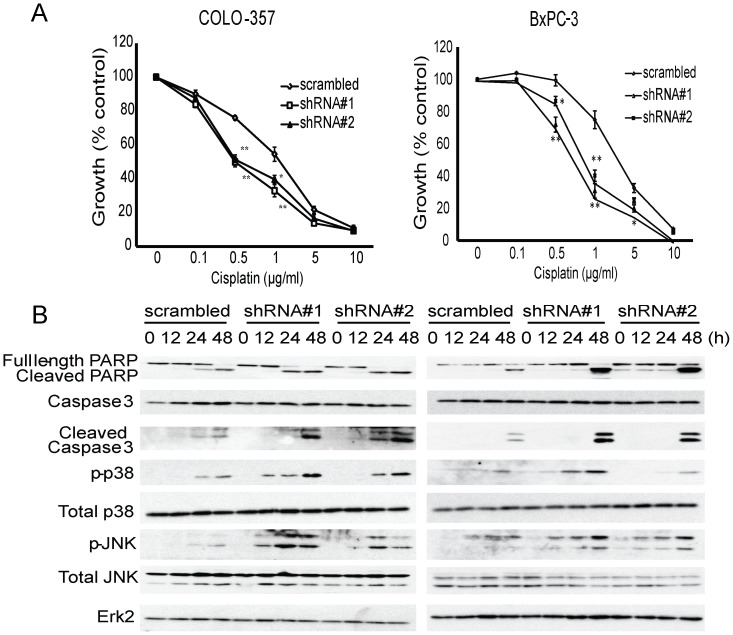
Effects of DUSP1 knockdown on cisplatin response of human pancreatic cancer cells. BxPC-3 and COLO-357 cells were stably transduced with lentivirus expressing shRNA against scramble control or DUSP1. (A) Cells were incubated for 48 h in the absence or presence of varying concentrations of gemcitabine, and MTT assays were performed. Data are the means ± SEM of 3 experiments. *p<0.05; **p<0.01, compared with control. (B) BxPC-3 and COLO-357 cells were incubated with 2 µg/ml cisplatin for the indicated times, and immunoblotting was conducted.

Considering that gemcitabine may be used in conjunction with cisplatin or 5-fluorouracil to treat locally advanced or metastatic PDAC [26], [27], we next sought to determine whether targeting DUSP1 would have similar sensitizing effects on other chemotherapeutic agents besides gemcitabine. To this end, BxPC-3 and COLO-357 cells stably expressing shRNA against DUSP1 or scramble control were treated with various concentrations of cisplatin. In both BxPC-3 and COLO-357 cells, DUSP1 knockdown decreased of the IC50 from 2 to 0.5 µg/ml, and also potentiated cisplatin-induced apoptotic cell death, as evidenced by increased levels of cleaved PARP and cleaved caspase 3 (Fig. 4A, 4B). Higher levels of cisplatin-induced phospho-p38 MAPK and phospho-JNK were also noted in cells with DUSP1 knockdown (Fig. 4B), and similar results were obtained with ASPC-1 cells (data not shown). Taken together, these results suggest that interrupting the negative feedback on p38 MAPK and JNK signaling by suppressing DUSP1 could enhance apoptosis and improve the chemosensitivity of pancreatic cancer to multiple chemotherapeutic agents.

### Knockdown of DUSP4, Another Nuclear DUSP, Fails to Enhance Chemosensitivity

**Figure 5:**
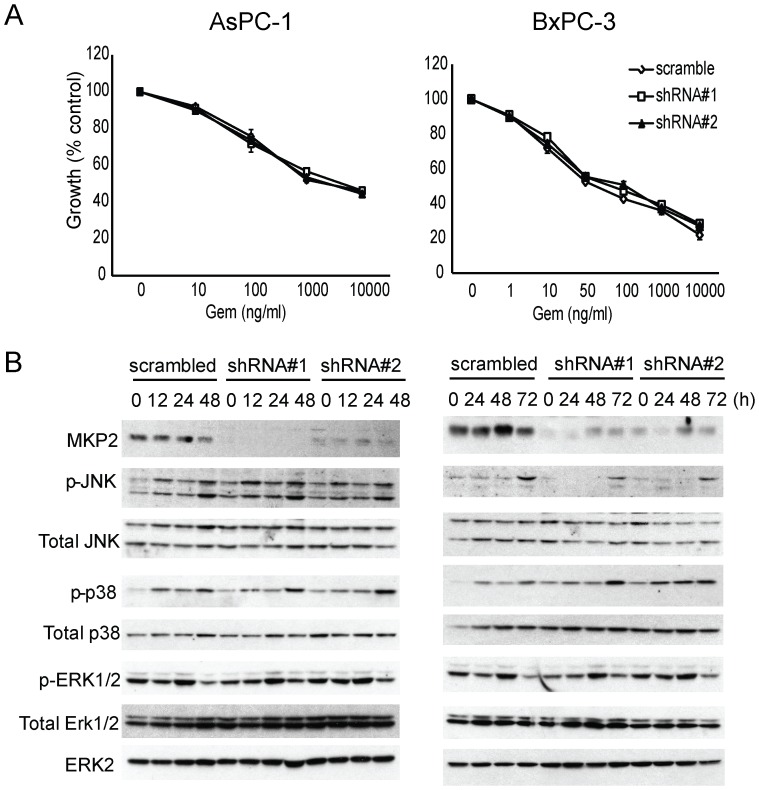
Knockdown of MKP2 does not affect JNK/p38 MAPK signaling activity or pancreatic cancer chemosensitivity to gemcitabine. AsPC-1 and BxPC-3 cells were stably transduced with lentivirus expressing shRNA against scramble control or MKP2. (A) Cells were incubated for 48 h in the absence or presence of varying concentrations of gemcitabine, and MTT assays were performed. (B) AsPC-1 and BxPC-3 cells were incubated for the indicated times with 100 ng/ml and 10 ng/ml gemcitabine, respectively, and immunoblotting was conducted. Data are the means ± SEM of 3 experiments.

We next sought to determine whether enhanced chemosensitivity is unique to DUSP1 silencing or a common feature of targeting any DUSP family members. Accordingly, we chose to knockdown DUSP4/MKP-2, due to its similarity to DUSP1 in terms of subcellular localization and substrate preference [11]. AsPC-1 and BxPC-3 cells were stably transduced with lentivirus encoding shRNA against DUSP4 or non-targeting scramble control. Successful silencing of DUSP4 was confirmed with immunoblotting (Fig. 5B). However, DUSP4 knockdown failed to affect MAPK activation or the response to gemcitabine in either cell line (Fig. 5A, 5B). Thus, DUSP1 is a potential therapeutic target for potentiating stress-activated MAPKs signaling and enhancing gemcitabine chemosensitivity, which is not necessarily a shared characteristic of all DUSP family members.

### Gemcitabine and DUSP1 Knockdown Combine to Prolong Survival, Attenuate Tumor Angiogenesis and Proliferation, and Enhance Apoptosis

**Figure 6:**
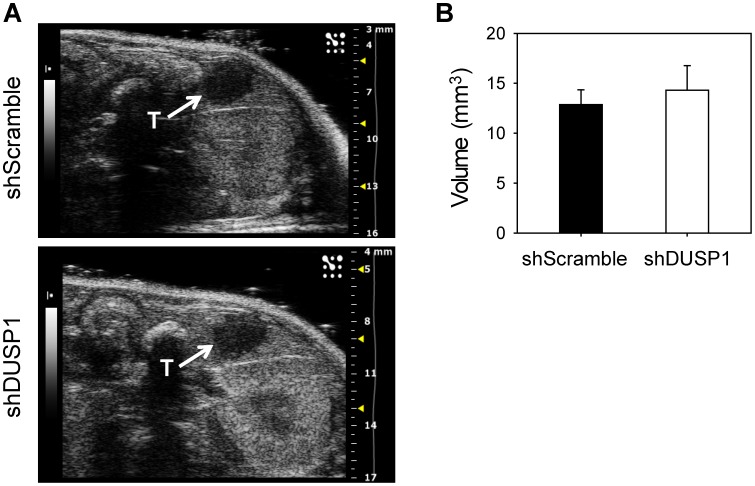
Quantitation of orthotopic tumors. COLO-357 cells were stably transduced with lentivirus expressing Tet-inducible control shRNA (shScramble) or DUSP1-targeting shRNA (shDUSP1). Cells were injected into the pancreata of immunodeficient mice, and 15 days later, tumors were imaged using a Vevo2100 high-resolution ultrasound. (A–B) Representative high-resolution ultrasound images (A) and quantitation of tumor volumes using 3D abdominal scans (B) show that prior to Dox or gemcitabine treatments, shScramble and shDUSP1 tumors (T, indicated by arrows) were similar in size. Data in (B) are the means ± SEM.

**Figure 7:**
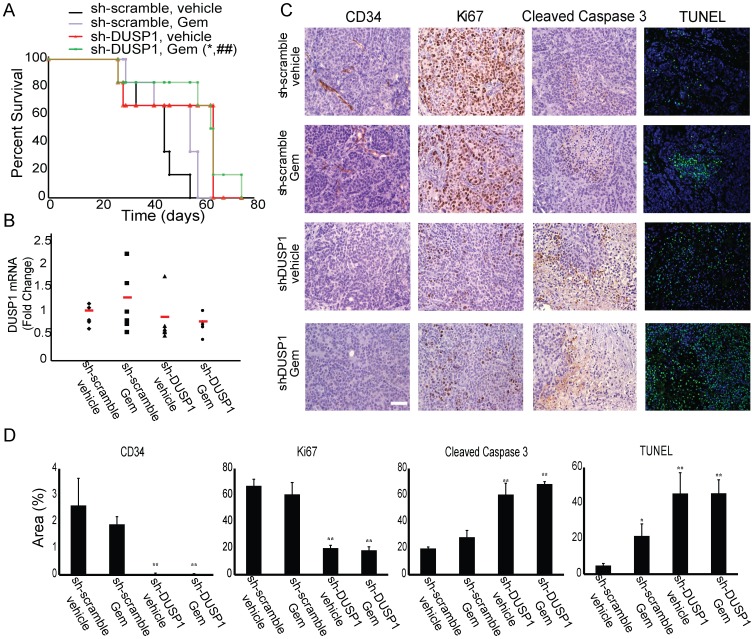
Effects of combining gemcitabine with DUSP1 knockdown on animal survival and tumor growth. Immunodeficient mice carrying orthotopic tumors derived from COLO-357 cells which stably express doxycycline-inducible shRNA against scramble control or DUSP1 were given doxycycline in the drinking water and treated with vehicle control (saline) or gemcitabine (50 mg/kg, i.p., twice weekly). (A) Kaplan-Meier survival. *p<0.05, compared with scramble treated with gemcitabine; ^##^p<0.01, compared with scramble treated with saline. (B) Q-PCR measurement of DUSP1 mRNA levels. (C) TUNEL staining and immunohistochemical analysis of CD34, Ki67, and cleaved caspase 3. Scale, 20 µm. (D) Quantification. Data are the means ± SEM of 3 experiments. *p<0.05; **p<0.01, compared with scramble treated with saline.

We next sought to evaluate the effect of inhibiting DUSP1 on gemcitabine chemosensitivity in an orthotopic mouse model. To study the therapeutic potential of targeting DUSP1 in fully established pancreatic tumors, we stably transduced COLO-357 human pancreatic cancer cells with lentivirus expressing doxycycline-inducible shRNA against DUSP1 or a non-targeting scramble control, before injecting the cells into the pancreas of immunodeficient mice in a TET-OFF state. Two weeks later, cells expressing doxycycline-inducible shRNA against DUSP1 or scramble control formed tumors of similar size, which could be easily palpated. All the mice were imaged on day 15 post-surgery, using a high resolution ultrasound, and tumor volumes were calculated based on acquired 3-D images, confirming that both groups formed tumors of equal volume (Fig. 6). Starting at day 18 post-surgery, doxycycline was continuously administered in the drinking water, and mice were randomized into 2 groups to receive vehicle or gemcitabine (50 mg/kg, intraperitoneal injection, twice weekly). Gemcitabine alone or DUSP1 silencing alone failed to prolong animal survival (Fig. 7A). However, comparison of the shRNA-scramble/gemcitabine group and the shRNA-DUSP1/gemcitabine group revealed that DUSP1 knockdown generated a significant survival advantage in the presence of gemcitabine (p = 0.037) (Fig. 7A). The increase in survival time was even greater when comparing the shRNA-DUSP1/gemcitabine group with the shRNA-scramble/vehicle group (p = 0.009), suggesting that DUSP1 silencing enhanced gemcitabine sensitivity *in vivo* (Fig. 7A).

In agreement with *in vitro* findings, gemcitabine treatment increased DUSP1 mRNA levels in shRNA-scramble tumors. Most of the shRNA-DUSP1 tumors had relatively low levels of DUSP1, whereas one tumor showed high DUSP1 levels, possibly due to shorter doxycycline exposure or contamination with adjacent non-tumor tissue when harvesting the sample. These results suggest that doxycycline successfully induced shRNA expression. However, due to high variability among each mouse, the difference in DUSP1 levels among different groups was not statistically significant (Fig. 7B).

To evaluate the effects of DUSP1 silencing and gemcitabine treatment on tumor angiogenesis, proliferation, and apoptosis, tumor tissues were next analyzed by immunohistochemical staining for CD34 (angiogenesis) and Ki67 (proliferation), as well as by terminal deoxynucleotidyl transferase dUTP nick end labeling (TUNEL) and cleaved caspase 3 immunoreactivity, both of which are markers of apoptosis. Gemcitabine slightly attenuated CD34 and Ki67 signals, while DUSP1 knockdown had a marked inhibitory effect on CD34 staining and a more moderate but highly significant inhibitory effect on Ki67 staining (Fig. 7C, 7D). DUSP1 silencing also led to enhanced cleaved caspase 3 immunoreactivity and TUNEL signals, compared with scramble control tumors, indicating that there was an increase in apoptotic cell death following DUSP1 knockdown. The increase in caspase 3 cleavage and DNA fragmentation was even greater when combining DUSP1 silencing with gemcitabine (Fig. 7C, 7D). Thus, targeting DUSP1 *in vivo* led to suppressed angiogenesis and cancer cell proliferation, and enhanced gemcitabine-induced apoptosis.

## Discussion

JNK1, -2, and -3 are encoded by *MAPK8*, *MAPK9* and *MAPK10*, respectively, and alternative splicing gives rise to at least ten isoforms [9]. By contrast, p38 MAPK-α, -β, -γ and -δ are encoded by *MAPK14*, *MAPK11*, *MAPK12*, and *MAPK13*, respectively, and there are two alternatively spliced isoforms of *MAPK14* [9]. Globally, JNK and p38 MAPK stress activated pathways induce apoptosis in some cases but enhance survival in others, depending on cell type-specific differences, the intensity and duration of signaling, and the presence or absence of crosstalk with other signaling pathways [9].

**Figure 8:**
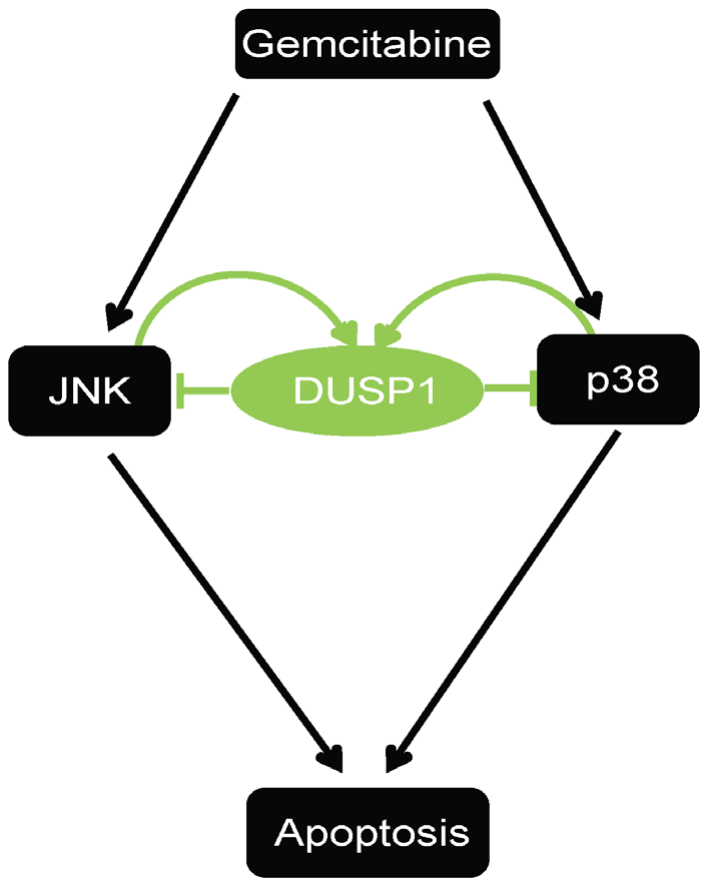
Model of the role of DUSP1 in promoting PDAC chemoresistance. Gemcitabine activates JNK and p38 MAPK (p38) signaling, which mediates apoptotic cell death. Activated JNK and p38 MAPK then upregulate DUSP1 transcription, which negatively modulates JNK and p38 MAPK signaling activity and attenuate gemcitabine-induced cell death. Inhibiting DUSP1 in combination with gemcitabine treatment significantly enhances chemosensitivity of pancreatic cancer cells.

In the current study we determined that gemcitabine activated JNK and p38 MAPK, thereby inducing apoptosis in PCCs. While the role of specific isoforms was not evaluated, gemcitabine-activated JNK and p38 MAPK signaling also induced DUSP1 transcription, as evidenced by increased DUSP1 mRNA levels and increased RNA polymerase II loading at DUSP1 gene body. Moreover, shRNA-mediated inhibition of DUSP1 enhanced gemcitabine-induced JNK and p38 MAPK activation and sensitized PDAC cells to gemcitabine and cisplatin, leading to decreased cell proliferation and increased PARP and caspase 3 cleavage. These results suggest that gemcitabine-mediated activation of JNK and p38 MAPK leads to the upregulation of DUSP1, which in turn contributes to a negative feedback loop that attenuates JNK and p38 MAPK activities, thereby interfering with the beneficial actions of gemcitabine on stress pathways and apoptosis (Fig. 8). These observations raise the possibility that DUSP1 can be a potential therapeutic target for enhancing PDAC sensitivity to multiple chemotherapeutic agents. Moreover, DUSP1 targeting leads to increased levels of p-ERK1 and p-ERK2 [15], and ERK activation in pancreatic cancer cells enhances gemcitabine chemoresistance [28]. Thus, gemcitabine-induced increases in JNK and p38 MAPK activities are crucial for its pro-apoptotic actions.

Downregulation of DUSP1 suppresses the expression of angiogenic factors, such as SH2D2A and VEGF-C in non-small-cell lung cancer (NSCLC) cells, and functional assays have confirmed the role of DUSP1 in promoting tumor angiogenesis [29]. Furthermore, in human NSCLC specimens, DUSP1 co-localizes with CD31-positive vascular structures, and a close correlation between increased VEGF-C and DUSP1 expression has been demonstrated [29]. These results support the concept that DUSP1 may play an important role in tumor angiogenesis. Although PDAC is characterized by marked desmoplasia and hypoperfusion, it also exhibits a high propensity to metastasize via hematogenous or lymphatic routes, even when the primary tumor is small [30]. Moreover, PDAC exhibits foci of micro-angiogenesis and overexpress multiple pro-angiogenic factors, and enhanced angiogenesis, high serum VEGF-A levels, and increased VEGFR-2 expression have been correlated with a worse prognosis in PDAC patients [30]. Together, these reports underscore the potential importance of aberrant angiogenesis in PDAC and implicate DUSP1 as contributing to this process.

Using doxycycline-inducible knockdown of DUSP1 in established orthotopic pancreatic tumors, in the present study we determined that combining gemcitabine with DUSP1 inhibition prolonged animal survival and enhanced apoptotic cell death, compared with gemcitabine alone. In addition, DUSP1 knockdown markedly attenuated PCC proliferation and tumor angiogenesis. Our use of immune deficient mice precludes an assessment of the consequence of DUSP1 inhibition on the immune system or on macrophage number and activation within the pancreatic tumor mass. Given that DUSP1 is known to modulate macrophage proliferation and activation [31], studies with syngeneic or genetically engineered mouse models of PDAC will be required to address this aspect of DUSP1 function in PDAC.

The mechanisms that lead to increased DUSP1 expression in PDAC are not known. It has been demonstrated, however, that hypoxia can upregulate DUSP1 transcription [14], and PDAC is a highly desmoplastic tumor with a markedly hypoxic microenvironment [5]–[7]. Moreover, in the presence of oxidative stress, E2F1 induces DUSP1 expression [13], and E2F1 is upregulated in PDAC as a consequence of RB dysfunction and excessive PI3K activation [32], [33]. Taken together with the present findings, these observations suggest that PDAC may be “primed” to exhibit increased DUSP1 activation in response to gemcitabine, and suggest that targeting DUSP1 in PDAC could enhance the beneficial actions of gemcitabine by promoting apoptosis and suppressing pancreatic cancer cell proliferation and tumor angiogenesis.

## Materials and Methods

### Ethics Statement

All animal studies were approved by the Institutional Animal Care and Use Committee of Indiana University (Permit Number: 10108). All animal studies described were conducted in accord with accepted standards of humane animal care and all efforts were made to minimize suffering.

### Cell Culture

AsPC-1 and BxPC-3 human pancreatic cancer cells were obtained from the American Type Culture Collection (Manassas, VA). COLO-357 cells were a gift from Dr. R. Metzger at Duke University, and were originally placed in culture by Morgan, *et al*., [34] from a patient with metastatic PDAC. They have been used extensively [15], [35], [36], and were authenticated by chromosomal analysis. AsPC-1 and BxPC-3 cells were grown in RPMI 1640, and COLO-357 cells were grown in DMEM. Unless otherwise specified, media were supplemented with 5% fetal bovine serum (FBS), 100 units/ml penicillin, and 100 µg/ml streptomycin (complete medium).

### 3-(4,5-Dimethylthiazol-2-yl)-2,5-diphenyltetrazolium Bromide (MTT) Assay

MTT assay was performed as described [35].

### Immunoblotting

Immunoblotting was done as described previously [35] using antibodies against the following antigens: PARP, Caspase-3, Cleaved Caspase-3 (Asp175), phospho-p38 MAPK (Thr180/Tyr182), p38 MAPK, phospho-JNK (G9), and phospho-c-Jun (Ser63) from Cell Signaling Technology, Danvers, MA; DUSP1(C-19), MKP2(S-18), JNK (FL) and ERK2 (C-14), from Santa Cruz Biotechnology, Santa Cruz, CA. Horseradish peroxidase-conjugated anti-mouse and anti-rabbit secondary antibodies were from BioRad, Hercules, CA.

### Reverse Transcription and Real-time Quantitative PCR

Total RNA was extracted using RNeasy purification kit (Qiagen, Valencia, CA). Total RNA (1 µg) was reverse transcribed using High-Capacity cDNA Reverse Transcription Kit. Taqman quantitative real-time PCR (Q-PCR) was carried out on an ABI Prism 7300 machine, and analyzed using a StepOnePlus Real-Time PCR system, all from Applied Biosystems, (Carlsbad, CA). All probes were pre-designed and obtained from Applied Biosystems. 18S was used as internal control. Gene expression levels were calculated using the relative ΔCt method [37].

### Chromatin Immunoprecipitation

BxPC-3 and COLO-357 cells were cross-linked with 1% formaldehyde for 10 min at 37°C. The cells were then rinsed with cold PBS, harvested and lysed with 1% SDS, 10 mM EDTA, and 50 mM Tris-HCl (pH 8.1) containing a protease inhibitor cocktail (Roche, Indianapolis, IN). Chromatin fragmentation was subsequently achieved by two sequential sonications (10 min each) using 30 sec on/off cycles and a Bioruptor sonicator (Diagenode, Denville, NJ) at the highest intensity. The soluble chromatin was diluted in buffer containing 1% Triton, 2 mM EDTA, 150 mM NaCl, and 20 mM Tris-HCl (pH 8.1) and added into Dynal magnetic beads (Qiagen, Valencia, CA) which were pre-incubated with anti-RNA polymerase II antibody (Abcam, Cambridge, MA) or control IgG. After 24 h, beads were washed and immune complexes were eluted using 100 ml of 1% SDS and 0.1 M NaHCO_3_ [38]. Samples were incubated overnight at 65°C to reverse cross-linking, and DNA was purified using PCR purification kit (Qiagen, Valencia, CA). Fold enrichments were determined using SYBR green Q-PCR (Applied Biosystems, Carlsbad, CA) according to the manufacturer’s protocol. The following primers were used for Q-PCR: DUSP1 gene body forward, TGTGGGCAACATTCCTGTAA; DUSP1 gene body reverse, CAAAGGATGGCACAGGATTT.

### Lentivirus-mediated Delivery of shRNA

The pTRIPZ lentiviral vectors for DUSP1 (V3THS_407291, V2THS_160994) and non-silencing shRNA control (RHS4743), and the pGIPZ lentiviral vectors for MKP2 (V3LHS_333999, V3LHS_334001) and non-silencing shRNA control (RHS4348) were purchased from Open Biosystems, Huntsville, AL. Packaging was performed using a second generation plasmid transfection system as previously described [36]. After infecting AsPC-1, BxPC-3, and COLO-357 cells with lentivirus in the presence of 8 µg/ml polybrene (Sigma-Aldrich, St Louis, MO), cells were selected with 6 µg/ml puromycin. For cells transduced with pTRIPZ lentivirus, DUSP1 levels were assessed by immunoblotting 72 h following the addition of 2 µg/ml doxycycline. Both puromycin and doxycycline were from Sigma-Aldrich (St Louis, MO).

### Orthotopic Implantation of Tumor Cells and Treatment Schedule

Twenty four 6- to 8-week-old male NOD/SCID/IL-2Rg^null^ mice were obtained from the In Vivo Therapeutics Core of Indiana University Simon Cancer Center. COLO-357 cells (0.5×10^6^) were stably transduced with pTRIPZ/sh-scramble or pTRIPZ/sh-DUSP1, suspended in 50 µl sterile PBS, and injected into the subcapsular region of the pancreas [39]. Tumors were imaged on day 15 post-surgery, using a Vevo2100 high resolution ultrasound (Visual Sonics Inc., Toronto, Canada), and tumor volumes were calculated based on acquired 3-D abdominal scans, using Vevo2100 System software (Visual Sonics). On day 18 post-surgery, mice were started on drinking water supplemented with 2 mg/ml doxycycline and 2% sucrose, and further randomized into two treatment groups: (1) vehicle control (saline); (2) 50 mg/kg gemcitabine (Biotang, Waltham, MA), intraperitoneal injection, biweekly. Individual mice were sacrificed when moribund according to the IACUC guidelines. Survival time was recorded, and tumor tissues were collected for further analysis. RNA was extracted from pancreatic tumors as previously described [40] using pre-cooled RNA extraction buffer consisting of 5 M guanidium thiocyanate, 50 mM Tris-HCl (pH 7.5), 2.5 mM EDTA, and 8% β-mercaptoethanol.

### Immunohistochemistry

Orthotopic tumors were fixed in 10% formalin and embedded in paraffin. H&E staining and immunohistochemistry were performed using 5 µm thick sections [41]. The following antibodies were used: Ki67 (Novacastra Leica Microsystems, Buffalo Grove, IL), cleaved caspase 3 (Asp175, Cell Signaling Technology, Danvers, MA), and CD34 (MEC 14.7, Abcam, Cambridge, MA). Sections were incubated in HRP-labeled secondary antibody and staining was detected by DAB (Dako, Carpinteria, CA). TUNEL assay was performed using the In Situ Cell Death Detection POD Kit (Roche, Indianapolis, IN) according to the manufacturer’s protocol. Images were taken using an Olympus BX60 microscope (Olympus, Center Valley, PA) equipped with a QImaging EXI Blue camera and ImagePro software (Media Cybernetics, Atlanta, GA).

### Statistical Methods

Statistical analysis was conducted using the GraphPad InStat software (version 3.00; GraphPad Software Inc., San Diego, CA). Significance was determined using one-way ANOVA, followed by the Dunnett test to compare all groups against the corresponding control group, and the Bonferroni test for specific pairwise comparisons. Statistical significance was taken as p≤0.05.
