# Correction: DUSP1 Is a Novel Target for Enhancing Pancreatic Cancer Cell Sensitivity to Gemcitabine

**DOI:** 10.1371/journal.pone.0108710

**Published:** 2014-09-15

**Authors:** The PLOS ONE Staff

Due to an error in the preparation of Figure 5B, two of the panels in this figure are incorrect: The "Total JNK" panel for BxPC-3 cells (right) incorrectly duplicates the "Total JNK" panel for AsPC-1 cells (left) The "p-ERK1/2" panel for the BxPC-3 cells (right) incorrectly displays the "p-ERK1/2" panel for AsPC-1 cells (left). The authors apologize for these mistakes and are supplying a corrected Figure 5 and the raw blots for this figure. These errors do not affect the results and conclusions reported in the article.

**Figure 5:**
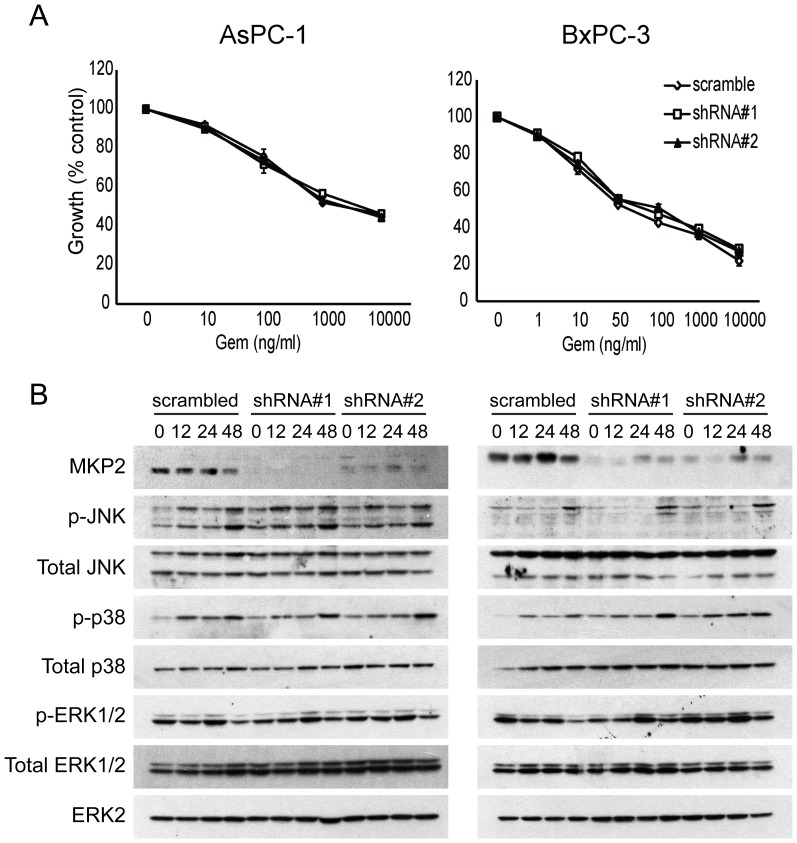
Knockdown of MKP2 does not affect JNK/p38 MAPK signaling activity or pancreatic cancer chemosensitivity to gemcitabine. AsPC-1 and BxPC-3 cells were stably transduced with lentivirus expressing shRNA against scramble control or MKP2. (A) Cells were incubated for 48 h in the absence or presence of varying concentrations of gemcitabine, and MTT assays were performed. (B) AsPC-1 and BxPC-3 cells were incubated for the indicated times with 100 ng/ml and 10 ng/ml gemcitabine, respectively, and immunoblotting was conducted. Data are the means ± SEM of 3 experiments.
