# Expression of Concern: DUSP1 Is a Novel Target for Enhancing Pancreatic Cancer Cell Sensitivity to Gemcitabine

**DOI:** 10.1371/journal.pone.0233098

**Published:** 2020-05-21

**Authors:** The PLOS ONE Editors

Following the publication and correction of this article [1, 2], the corresponding author notified the journal of additional concerns, prompting an editorial reassessment of this work. The following issues involving Figs 1B, 1C, 2A and 3B and the Results section have been raised since the Correction [2] was published:

- The same ERK2 data are reported in Figs 1B and 2A. The corresponding author indicated that the same ERK2 control panel applies to both figures, as they reported results using the same samples and blots. However, this was not indicated in the published article. The ERK2 panels in Fig 1B have been replaced with Total ERK panels in the revised figure available with this notice.
- The lanes 1–4 in Fig 1B Phospho-JNK and Phospho-c-Jun panels appear similar, and according to the corresponding author the two panels report the same blank area of film rather than the indicated control data. Fig 1B has been updated to include the correct control data in these panels.
- The corresponding author has indicated that in Fig 1B, the PARP, Caspase, and Total p38 panels of AsPC-1 cells, the PARP, Caspase, and Phospho-p38 panels of BxPC-3 cells, and the PARP and Caspase panels of the COLO-357 cells originate from a different experiment than the rest of the panels presented in the figure.
- The PARP panel shown in Fig 1B for AsPC-1 appears similar to lanes 1–4 of the PARP panel in Fig 3B. Fig 3B indicates that these data reflect results of experiments in which cells were stably transduced with a scramble control shRNA construct, whereas this treatment is not indicated for the Fig 1B experiment.
- The ERK2 panel in Fig 1C is mislabelled. The correct notation for this panel is Total Caspase 3. The correct ERK2 panel is included in the revised figure available with this notice.
- There is an error in Fig 2A. The DUSP1 panel for AsPC-1 cells is from a different experiment than the ERK2 panel. Therefore, in the updated figures all panels have been replaced with results originating from the same representative experiment.
- The corresponding author noted that there is an error in the sixth sentence of the second paragraph of the Results. The correct sentence is: “By contrast, AsPC-1 cells were more resistant to gemcitabine, as evidenced by the absence of p38 MAPK and JNK activation and the appearance of cleaved caspase 3 and cleaved PARP only when the concentration of gemcitabine was increased to 100 ng/mL (Fig 1B).”

**Fig 1:**
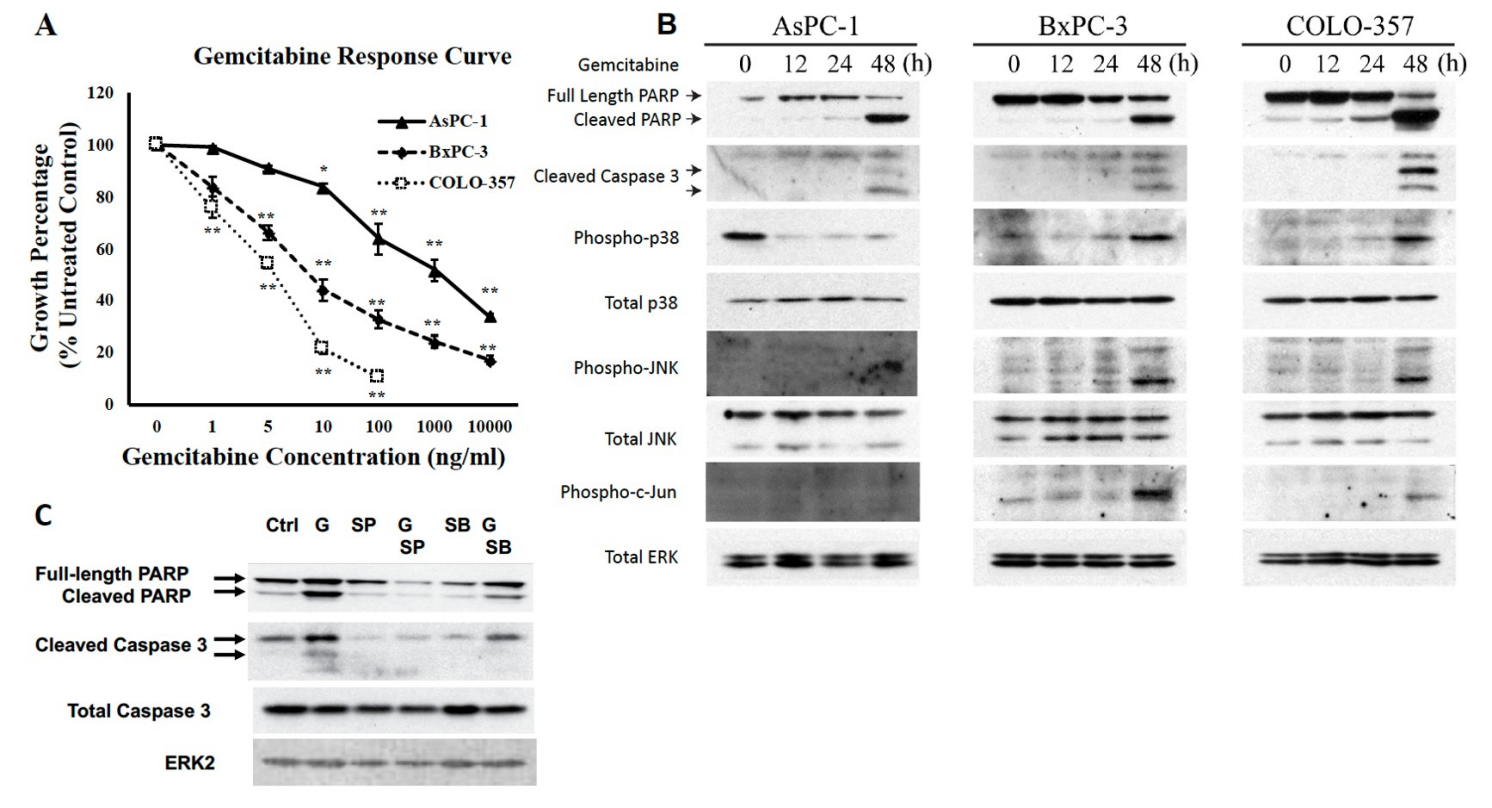
Effects of gemcitabine on human pancreatic cancer cells. (A) AsPC-1, BxPC-3, and COLO-357 cells were incubated for 48 h in the absence or presence of varying concentrations of gemcitabine, and MTT assays were performed. Data are the means ± SEM of 3 experiments. *p<0.05; **p<0.01, compared with control. (B) AsPC-1, BxPC-3, and COLO-357 cells were incubated for the indicated times with 100 ng/ml, 10 ng/ml, and 5 ng/ml gemcitabine, respectively, and analyzed by immunoblotting. All blots shown in panel B are from a single representative experiment for each cell line. (C) BxPC-3 cells were incubated for 48 h with 10 ng/ml gemcitabine (G), in the absence or presence of 10 μM SB203580 (SB) or 10 μM SP600125 (SP), and analyzed by immunoblotting.

**Fig 2:**
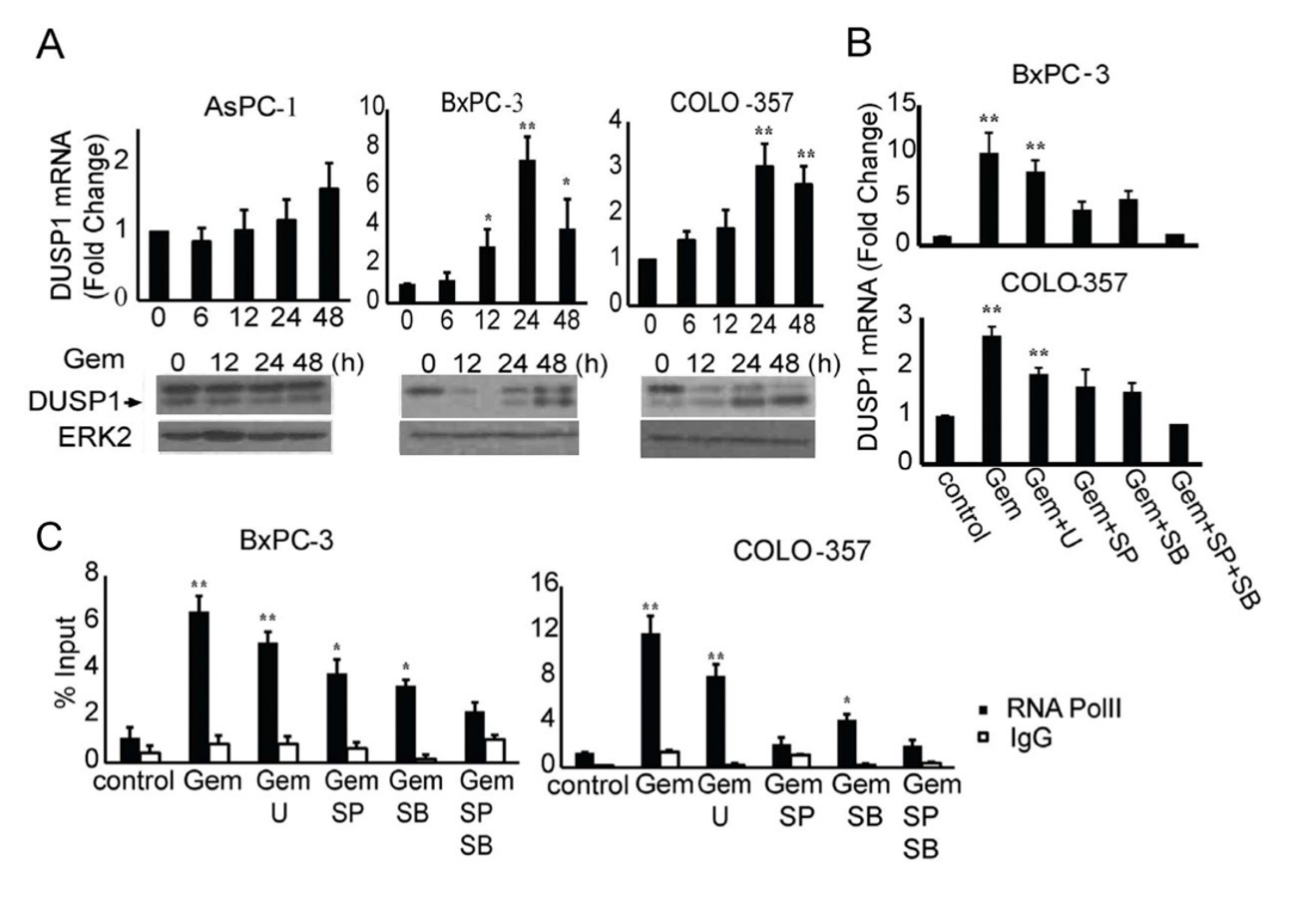
Gemcitabine induces DUSP1 transcription through JNK and p38 MAPK signaling. (A) AsPC-1, BxPC-3, and COLO-357 cells were incubated for the indicated times with 100 ng/ml, 10 ng/ml, and 5 ng/ml gemcitabine, respectively, and DUSP1 levels were assessed by Q-PCR. Additionally, immunoblotting of cell lysates from AsPC-1, BxPC-3, and COLO-357 cells that were incubated for the indicated times with 100 ng/ml, 10 ng/ml, and 5 or 10 ng/ml gemcitabine, respectively, revealed that gemcitabine increased DUSP1 protein levels in BxPC-3 and COLO-357 cells, but not in AsPC-1 cells. In the example shown COLO-357 cells were incubated with 10 ng/ml gemcitabine. (B,C) BxPC-3 and COLO-357 cells were incubated for 24 h with 10 ng/ml and 5 ng/ml gemcitabine, respectively, in the absence or presence of 10 μmol/L U0126, 10 μmol/L SP600125, 10 μmol/L SB203580, or both SP600125 and SB203580, and Q-PCR (B) and RNA polymerase II ChIP followed by Q-PCR for DUSP1 gene body region (C) were performed. All RNA-related data are the means ± SEM of 3 experiments. *p<0.05; **p<0.01, compared with control.

The corresponding author has provided corrected Figs 1 and 2 with replacement panels for Fig 1B (PARP lanes 1–12, Cleaved Caspase 3 lanes 1–12, Phospho-p38 lanes 5–8, Total p38 lanes 1–4, Phospho-JNK lanes 1–4, Phospho-c-Jun lanes 1–4) and Fig 2A (DUSP1, ERK2). Per the corresponding author, the replacement panels reflect the correct data from the original experiments and all panels in the updated figures are from the same representative experiment.

The raw data underlying the updated versions of Figs 1 and 2 are provided in S1–S3 Files below. The original data supporting other results in this article are no longer available.

The *PLOS ONE* Editors issue this Expression of Concern to notify readers of the above concerns and relay the supporting data and updated figures provided by the corresponding author.

## Supporting information

S1 File Original blot images underlying the Phospho-p38, Total p38, pERK^1^/_2_, total ERK^1^/_2_, Phospho-JNK, Total JNK^1^/_2_, cleaved PARP, total Caspase 3, cleaved Caspase 3, and Phospho-c-Jun, panels for AsPC-1, BxPC-3, and COLO-357 in the revised version of Fig 1B. (PDF)

S2 File Scan of original blot images underlying the Full-length and cleaved PARP, Cleaved Caspase 3, Total Caspase 3, and ERK2 panels presented in the revised version of Fig 1C. (JPEG)

S3 File Scan of original blot images underlying the DUSP1 and Erk2 panels for AsPC-1, BxPC-3, and COLO-357 in the revised versions of Fig 2A. (TIF)
