# Supplementary figures and images for: Expression of Concern: DUSP1 Is a Novel Target for Enhancing Pancreatic Cancer Cell Sensitivity to Gemcitabine
Source: PLoS One. 2020 May 21;15(5):e0233098. doi: 10.1371/journal.pone.0233098 (PMC7241812; doi:10.1371/journal.pone.0233098)

# ASPC-1

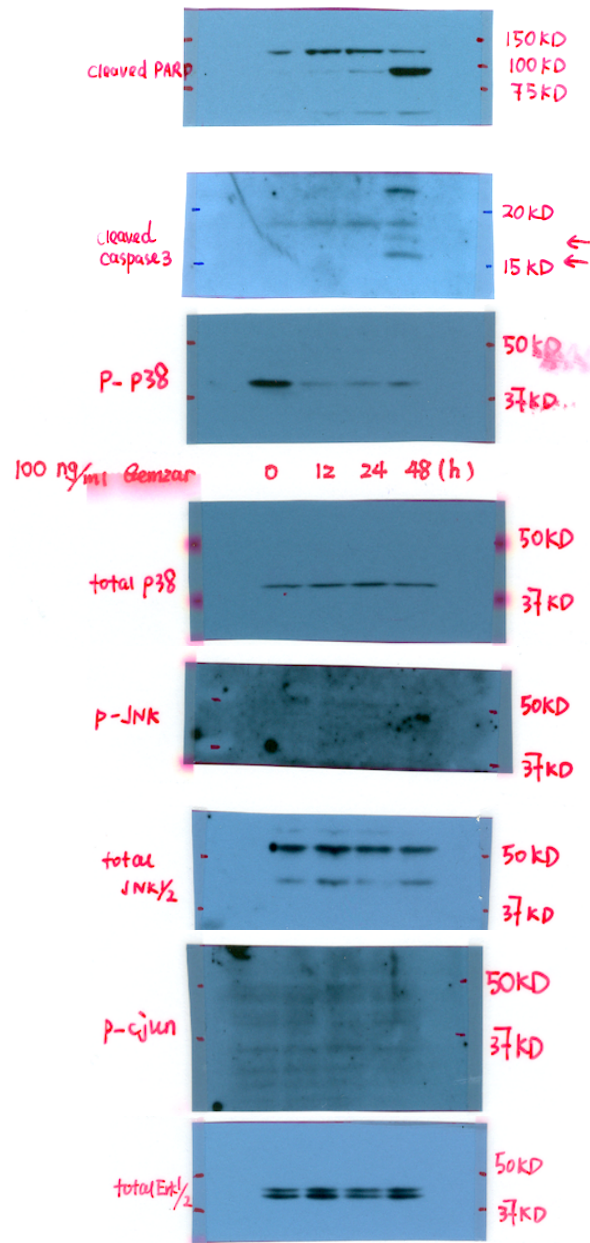

# BxPC-3

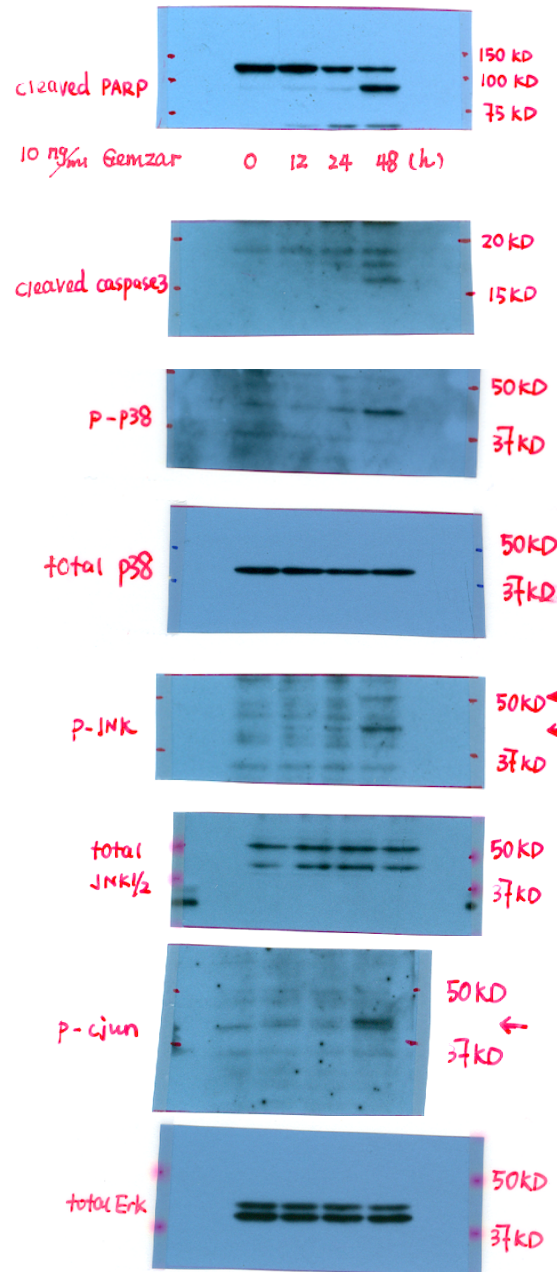

# COLO-357

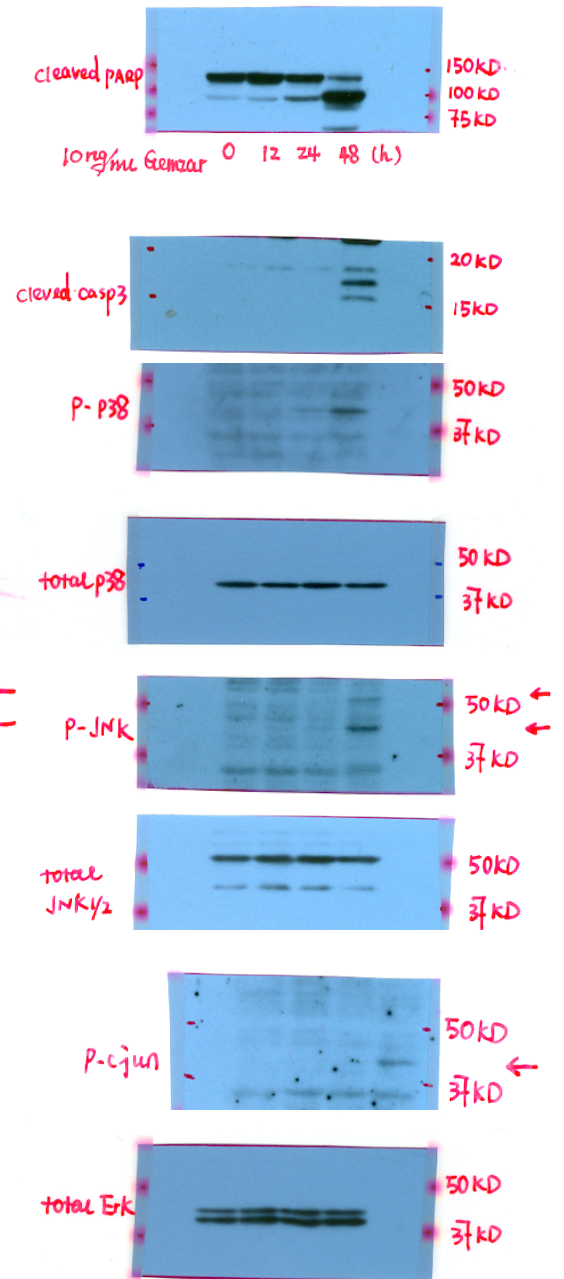

Supplement: S1 File — (PDF) [file pone.0233098.s001.pdf]

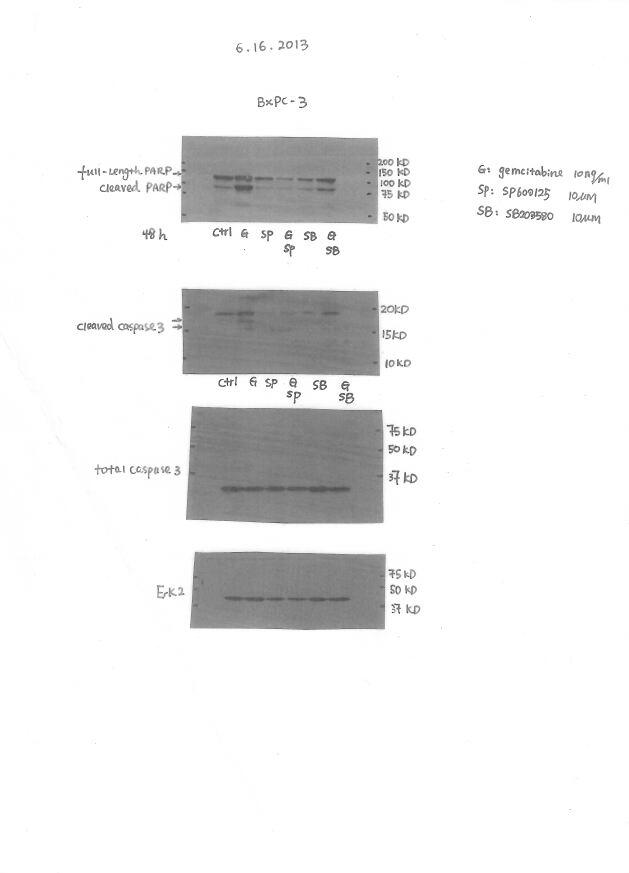

Supplement: S2 File — (JPEG) [file pone.0233098.s002.jpeg]

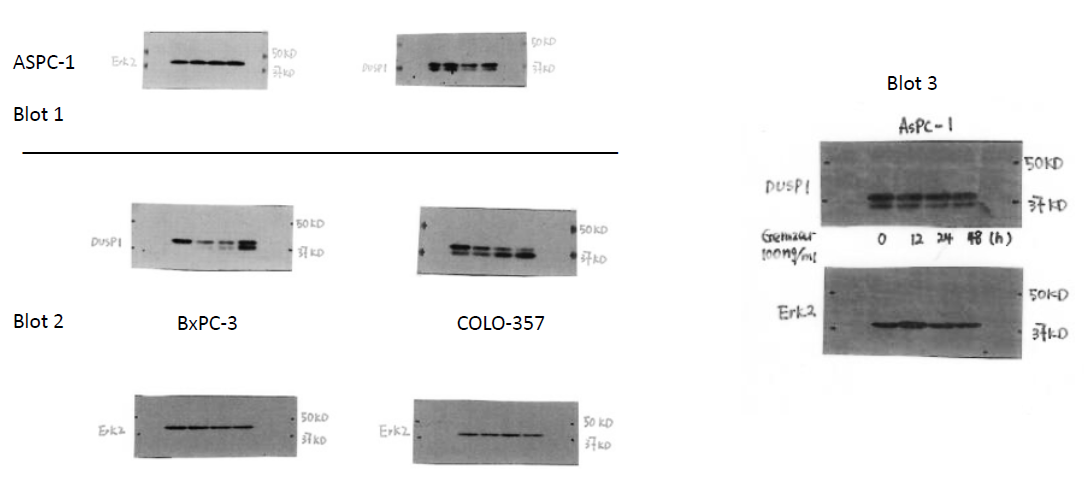

Supplement: S3 File — (TIF) [file pone.0233098.s003.tif]
